# Positive Psychological Factors Relate to Domain-Specific Cognition and Daily Functioning in Middle-Aged and Older Adults with HIV

**DOI:** 10.1007/s10461-025-04636-8

**Published:** 2025-03-03

**Authors:** Lillian Ham, Maulika Kohli, Bin Tang, Igor Grant, David J. Moore

**Affiliations:** 1 San Diego Joint Doctoral Program in Clinical Psychology, San Diego State University, University of California, 220 Dickinson Street, San Diego, CA 92103 USA; 2 HIV Neurobehavioral Research Program, University of California San Diego, 220 Dickinson Street, Suite B (8231), San Diego, CA 92103 USA; 3 VA Puget Sound Healthcare System, Seattle Division, Seattle, WA USA; 4 Department of Psychiatry, University of California San Diego, La Jolla, CA USA

**Keywords:** Positive psychology, Psychological resilience, Social support, Neurocognitive disorders, Activities of daily living, Aging

## Abstract

Understanding positive psychological factors (PPFs; internal strengths, socioemotional support) that promote optimal health outcomes among people with HIV (PWH) is increasingly important. 122 PWH and 98 people without HIV (PWoH) were included in multivariable regressions, testing interactions between HIV status and PPFs on 7 cognitive domains and 2 daily functioning outcomes, controlling for depressive symptoms. Overall, higher internal strengths were related to better learning and memory, whereas higher socioemotional support was related to better processing speed and psychomotor speed. PWH had a stronger positive relationship between PPFs and verbal fluency than PWoH. Overall, higher internal strengths were associated with fewer daily functioning impairments; however, only PWoH showed fewer functional declines with higher socioemotional support. PWH may require greater socioemotional support to accommodate an increased burden of comorbidities leading to functional difficulties. Among PWH, bolstering PPFs may improve cognition; however, internal strengths may particularly benefit daily functioning.

**Supplementary Information:**

The online version contains supplementary material available at 10.1007/s10461-025-04636-8.

## Resumen

Entender factores de la Escala de Funcionamiento Psicológico Positivo (EFPP), como fuerza interna y apoyo socioeconómico, que promueven la salud óptima de individuos con VIH es importante. 122 individuos con VIH y 98 individuos sin VIH fueron incluidos en un análisis que utilizó una regresión multivariada para explorar interacciones entre estado de VIH y los factores de la EFPP, sobre 7 habilidades cognitivas y 2 medidas de actividades de la vida diaria, considerando síntomas depresivos reportados. Mayor fuerza interna fue relacionada con mejores niveles de memoria y aprendizaje, mientras que mejor apoyo socioeconómico se relacionó con velocidad de procesamiento y velocidad psicomotriz. Entre los individuos con VIH se observó una asociación positiva más fuerte entre factores de la EFPP y fluidez verbal, comparados con individuos sin VIH. Una mayor fuerza interna se relacionó con menores impedimentos en actividades de la vida diaria; pero, solo en individuos sin VIH se observó una asociación entre mejor funcionamiento en actividades de la vida diaria y mayor apoyo socioeconómico. Puede ser que los individuos con VIH necesitan mayor apoyo socioeconómico para acomodar una mayor cantidad de condiciones médicas secundarias que resultan en menos capacidad para completar actividades de la vida diaria. Entre individuos con VIH, mejorar factores psicológicos positivos puede resultar en mejoras a su habilidad cognitiva; pero, la fuerza interna puede brindar mayores beneficios en su funcionamiento diario.

## Introduction

Despite vast improvements in HIV treatment, people with HIV (PWH) remain at elevated risk for neurocognitive impairment (NCI) [1, 2] and functional decline relative to the general population. Pathophysiological processes [3] (e.g., chronic inflammation, immune dysfunction) associated with HIV are purported to drive abnormal aging trajectories (i.e., premature, accelerated) [4–8] whereby PWH are less likely than people without HIV (PWoH) to preserve cognition and functional independence in older age. NCI among PWH is associated with unemployment and functional difficulties, particularly in more complex tasks such as medication management and driving [9–11]. Functional decline remains high among PWH at up to 75% [12, 13], which is nearly three times the rate in PWoH with similar demographics and risk factors. The investigation of modifiable factors that promote successful aging and resilience among PWH [10] has increased in urgency as more than half of PWH in the U.S. are 50 years or older [14]. With the rise in positive psychology and positive psychiatry, there have been increased efforts to identify psychosocial variables that may be leveraged to improve health and well-being for PWH.

Positive psychological factors (also referred to as psychological reserve [15] or reserve capacity [16, 17]) are strengths-based, non-cognitive characteristics related to well-being and are independent of negative affect [18–20] and psychopathology [21]. Positive psychological factors (hereafter, PPFs) may lead to more successful aging by allowing an individual to cope with problems that emerge with aging [22]. In support of this hypothesis, PPFs have been associated with better cognition and longer lifespan [23] among older adults. For example, higher levels of gratitude are positively associated with better global cognitive status [24] and greater perceived control is positively associated with better memory, processing speed, and verbal intelligence [25]. Emotional support is also a significant predictor of better cognition over time [26]. PPFs may even predict cognitive trajectories decades later. For example, higher levels of grit (i.e., perseverance toward long-term goals) in adolescence has been shown to predict better learning and memory in late life, suggesting that non-cognitive positive factors may be protective against age-related declines [27].

Successful aging models have posited that older adults with chronic illnesses (e.g., HIV) and functional impairment can experience successful aging by practicing and promoting compensatory mechanisms such as PPFs [28, 29]. PPFs can buffer against the deleterious effects of stress on health [30] leading to NCI and functional impairment by increasing health promoting behaviors [31, 32], improving physiological functioning (e.g., decreased inflammation) and physical health [20, 33, 34], and recruiting additional positive resources [35–37]. In fact, PPFs have been associated with successful cognitive aging (defined as no NCI, functional impairment, or major depressive disorder) [38], better neurocognition [15, 39], and better daily functioning [15, 39] in PWH. In the absence of depression, PWH report similar levels of health-related quality of life and PPFs compared to PWoH [40]. Thus, bolstering PPFs may have optimal downstream health outcomes in PWH through multiple pathways.

Though PPFs and their relationship to cognition and daily functioning have been examined in the general population, few studies have compared these relationships between PWH and PWoH. Additionally, while findings with PPFs and health outcomes have been promising, most studies have included a heterogeneous mix of positive constructs without much specificity. Thus, the unique contribution of PPFs to cognition and daily functioning in PWH is currently unclear. An eight-factor structure [41] was proposed as a measure of multidimensional resilience for older PWoH, which included items from various internal (i.e., individual) and external (i.e., interpersonal) PPFs (e.g., optimism, social support). Internal and external PPFs have constituted independent latent factors among PWH. Rubtsova and colleagues (2018) [42] examined the psychosocial correlates of physical frailty and found two latent factors among measures of positive *and* negative psychological factors: (1) positive resources/outlook (i.e., grit, optimism, personal mastery, successful aging, depression [inverse], perceived stress [inverse], negative interactions [inverse]) and (2) support by others (i.e., social support, emotional support). While ‘support by others’ was negatively associated with physical frailty regardless of HIV status, ‘positive resources/outlook’ was negatively associated with physical frailty only in PWH. Ham and colleagues (2023) [43] supported a similar factor structure (i.e., one internal and one external latent factor) while considering only positive psychological measures: 1) internal strengths (i.e., positive attitudes toward aging, optimism, personal mastery, life satisfaction, grit) and 2) socioemotional support (i.e., social support, emotional support). Internal strengths increased over four years for PWH only, while socioemotional support remained stable in both PWH and PWoH. Both studies suggest that internal and external PPFs experienced by PWH may be unique; however, these groups of PPFs have yet to be examined in relation to cognition and daily functioning.

### The Current Study

This study aimed to fill this gap in research on specific PPFs and their relationship to cognition and daily functioning among PWH. Given that PWH face greater risk for NCI and functional impairment, and yet, display resilience and successful aging, PWH may have a greater reliance on PPFs [42, 43] to maintain optimal cognition and daily functioning. Thus, we hypothesized that (1) PWH will have stronger positive associations between PPFs and cognition compared to PWoH; and (2) PWH will have stronger negative associations between PPFs and daily functioning impairments compared to PWoH.

## Methods

### Participants

Both PWH and PWoH were recruited from the *Multi-Dimensional Successful Aging Among HIV-Infected Adults* study, which has been described in prior publications [38, 42, 43]. This longitudinal study was approved by the University of California, San Diego (UC San Diego) Institutional Review Board and conducted at the UC San Diego HIV Neurobehavioral Research Program (HNRP) and the UC San Diego Stein Institute for Research on Aging. Data were collected from 2013 to 2019. A written informed consent including potential risks and benefits was obtained from all participants after the study was explained to them by a trained staff member. Exclusion criteria were acute drug or alcohol intoxication on the day of testing (e.g., positive urine toxicology screen for illicit substances other than cannabis), significant neurological/neurodegenerative conditions unrelated to HIV (e.g., Alzheimer’s disease), and serious psychotic disorders (e.g., schizophrenia). Inclusion criteria were age 36 to 65 years, English fluency, and ability to provide informed consent. All participants were assessed for comorbid medical conditions (e.g., self-reported presence of hepatitis C, hyperlipidemia, diabetes) and psychiatric conditions (e.g., major depressive disorder [MDD], substance use disorder). The age range of participants, although middle-aged, is appropriate for the aims of the current study as prior work has shown that PWH experience premature aging, including earlier onset of NCI and functional decline relative to the general population. Community-dwelling adults in San Diego and surrounding areas were considered for inclusion.

### Measures

#### Positive Psychological Factors

Seven, self-report questionnaires were administered reflecting individual, positive psychological measures. Composite z-scores representing two groups of PPFs (internal strengths and socioemotional support) were calculated for the overall sample (i.e., both PWH and PWoH) by 1) generating z-scores for each positive psychological measure based on PWoH’s mean and standard deviation (SD):

$$\:z=\:\frac{\text{R}\text{a}\text{w}\:\text{s}\text{c}\text{o}\text{r}\text{e}\:{\text{-}}\:\text{M}\text{e}\text{a}\text{n}\:\text{s}\text{c}\text{o}\text{r}\text{e}\:\text{f}\text{o}\text{r}\:\text{P}\text{W}\text{o}\text{H}}{\text{S}\text{D}\:\text{f}\text{o}\text{r}\:\text{P}\text{W}\text{o}\text{H}}$$

and by 2) averaging z-scores across 5 measures of internal strengths (grit, personal mastery, optimism, positive attitudes toward aging, and life satisfaction) and 2 measures of socioemotional support (social and emotional support). Higher z-scores represented greater positive psychological functioning relative to PWoH’s scores. These composite factors were derived based on results from a published exploratory factor analysis [43] (Supplementary Table 1).

#### Neurocognitive Functioning

A comprehensive, gold-standard neuropsychological battery comprised of 7 cognitive domains (verbal fluency, executive functioning, processing speed, learning, memory, attention/working memory, and psychomotor speed) was administered. Raw test scores were converted to T-scores (*M* = 50, *SD* = 10) to create domain-specific (i.e., averaged tests within each domain) and global cognition scores (i.e., averaged scores across all domains) that were demographically adjusted for age, gender, race, and education. Details of the neuropsychological battery are described in Supplementary Table2.

#### Daily Functioning

Two frequently used self-report questionnaires in HIV to assess daily functioning were administered [56]. The modified Instrumental Activities of Daily Living (IADL) [57] questionnaire measured self-reported levels of independent functioning in 13 domains (e.g., financial management, medication management, bathing). Participants reported two ratings per functional domain: current (“Now”) vs. best functioning level (“Best”). Any declines from “Best” to “Now” counted as a functional “decline”. Higher count scores represented greater functional decline (total count range: 0–13).

The 34-item Patient’s Assessment of Own Functioning Inventory (PAOFI) [58] measured self-reported cognitive symptoms in daily life (e.g., memory, language and communication, sensory-perceptual, higher level cognitive functions). Items measured the frequency of impairment in a domain with Likert-type responses (“Almost always” to “Almost never”). Responses occurring with at least “Fairly often” or greater frequency counted as a functional “impairment”. Higher count scores represented greater cognitive symptoms (total count range: 0–34).

#### Depressive Symptoms

The 20-item Center for Epidemiologic Studies Depression Scale (CES-D) [59] measured self-reported depressive symptoms. Items measured the frequency of depressive symptoms experienced in the past week on a 0–3 scale, with higher scores reflecting greater depression (total sum range: 0–60).

### Statistical Analyses

Group differences by HIV status were compared using independent samples t-tests and chi-square tests of independence. CES-D total sum was log_10_ transformed to normalize its distribution. Objective neurocognitive outcomes included global and domain-specific T-scores, and daily functioning outcomes included number of self-reported functional declines (IADL) and cognitive symptoms (PAOFI). The effects of HIV status, PPFs, their interaction, and depressive symptoms were assessed in multiple linear regressions for continuous outcomes (i.e., global and domain-specific cognition T-scores) and negative binomial regressions for count outcomes (i.e., number of functional declines and cognitive symptoms) due to overdispersion (i.e., variance greater than the mean). Given the frequent comorbidity of HIV and depression, in addition to previous findings that depression is inversely but independently related to PPFs [18–21], depressive symptoms (i.e., log CES-D total sum) were included in all regression models. Significant interactions were followed up by simple main effects stratified by HIV status including relevant covariates.

Demographic factors (age, education, gender, race) were considered for inclusion in final models if there was a univariate association with daily functioning at *p* < 0.15 in negative binomial regressions. Neurocognitive T-scores were demographically adjusted for age, education, gender, and race (where possible); thus, demographic factors were not considered in linear regression models. Two-tailed tests with a significance level of 0.05 were conducted in JMP Pro 17.0.0 and IBM SPSS Statistics 28.01.01 (14).

## Results

### Participants

All 220 participants (122 PWH; 98 PWoH) considered for inclusion completed at least 4 of 5 positive psychological measures for internal strengths (missing one questionnaire: *n* = 12) and at least 1 of 2 positive psychological measures for socioemotional support (missing one questionnaire: *n* = 7). Due to minimal missing data and efforts to conserve statistical power, all participants were retained for analyses.

Participant sociodemographic factors and comorbidities by HIV status are summarized in Table 1. The total sample ranged in age from 36 to 65 years. Compared to PWoH (all *p-values* < 0.05), PWH had a greater proportion of male participants (84% v. 32%, $\:{\chi\:}^{2}$ = 7.07) and smaller proportion of White participants (54% v. 68%, $\:{\chi\:}^{2}$ = 4.67), though a majority identified themselves as White. PWH had slightly fewer mean years of education (14 v. 15, t = -3.27) and lower rates of employment (31% v. 74%, $\:{\chi\:}^{2}$ = 40.88). With regards to comorbidities, PWH had higher rates of hepatitis C (18% v. 4%, $\:{\chi\:}^{2}$ = 11.28), hypertension (44% v. 19%, $\:{\chi\:}^{2}$ = 15.69), and hyperlipidemia (41% v. 22%, $\:{\chi\:}^{2}$ = 8.67). PWH also had a greater severity of current depressive symptoms (17 v. 14, t = 4.10), and higher rates of current MDD (11% v. 0%, $\:{\chi\:}^{2}$ = 16.41), lifetime MDD (55% v. 19%, $\:{\chi\:}^{2}$ = 29.05), and lifetime substance use disorder (68% v. 38%, $\:{\chi\:}^{2}$ = 20.25). PWH had lower internal strengths than PWoH (-0.7 v. 0.0, t = -5.89), but not lower socioemotional support (-0.2 v. 0.0, t = -1.86; Table 2).

**Table 1:** Sociodemographic differences and comorbidities by HIV status

	PWH (*n* = 122)	PWoH (*n* = 98)	Test statistic	Effect size
*Demographics*				
Age (years)	50.7 (8.4)	51.1 (7.6)	t = -0.38	d = 0.05
Sex, N (% male)	102 (83.6)	31 (31.6)	$\:{\chi\:}^{2}$ = 7.07**	V = 0.18
Race/ethnicity, N (%)			$\:{\chi\:}^{2}$ = 4.67*	V = 0.15
White	66 (54.1)	67 (68.4)		
Hispanic	23 (18.9)	17 (17.3)		
Black	23 (18.9)	13 (13.3)		
Asian	1 (0.8)	0 (0.0)		
Other	9 (7.4)	1 (1.0)		
Education (years)	14.1 (2.4)	15.1 (2.2)	t = -3.27**	d = 0.45
Employed, N (%)	36 (30.5)	72 (73.5)	$\:{\chi\:}^{2}$ = 40.88***	V = 0.43
*Comorbidities*				
Hepatitis C, N (%)	22 (18.0)	4 (4.1)	$\:{\chi\:}^{2}$ = 11.28**	V = 0.21
Hypertension, N (%)	54 (44.3)	19 (19.4)	$\:{\chi\:}^{2}$ = 15.69***	V = 0.26
Hyperlipidemia, N (%)	50 (41.0)	22 (22.4)	$\:{\chi\:}^{2}$ = 8.67**	V = 0.20
Diabetes, N (%)	14 (11.5)	7 (7.1)	$\:{\chi\:}^{2}$ = 1.21	V = 0.07
*Psychiatric characteristics*				
Depressive sx (CES-D)	17 [13–23]	14 [12–17]	t = 4.10^a^***	d = 0.57
Current MDD, N (%)	12 (11.3)	0 (0.0)	$\:{\chi\:}^{2}$ = 16.41***	V = 0.24
LT MDD, N (%)	62 (54.9)	19 (19.4)	$\:{\chi\:}^{2}$ = 29.05***	V = 0.36
Current substance use dx, N (%)	4 (3.8)	1 (1.0)	$\:{\chi\:}^{2}$ = 1.74	V = 0.09
LT substance use dx, N (%)	78 (68.4)	37 (37.8)	$\:{\chi\:}^{2}$ = 20.25***	V = 0.31
*HIV characteristics*				
Current CD4 (c/µL)	638 [422–854]	--	--	--
Nadir CD4 (c/µL)	176 [46–329]	--	--	--
HIV duration (years)	18.4 [9.6–25.2]	--	--	--
On ART, N (%)	112 (94.9)	--	--	--
AIDS status, N (% AIDS)	75 (61.5)	--	--	--
Plasma viral load, N (% ≤ 50 c/mL)	100 (91.7)	--	--	--

Note.^a^ = test conducted with log10 transformed values, *p < 0.05, **p < 0.01, ***p < 0.001. Mean (standard deviation) shown for continuous variables, median [interquartile range] shown for count variables, and % for categorical variables; PWH = people with HIV; PWoH = people without HIV; sx = symptoms; CES-D = Center for Epidemiological Studies Depression Scale; LT = lifetime; MDD = major depressive disorder; dx = diagnosis; ART = antiretroviral therapy; psych. = psychological; d = Cohen’s d; V = Cramer’s V. *p*-values < 0.05 are bolded and denote statistical significance

**Table 2:** Positive psychological factors, neurocognition, and daily functioning by HIV status

	PWH	PWoH	Test statistic	Effect size
*Positive psych. factors*, *M (SD)*				
Internal strengths	-0.74 (1.04)	-0.01 (0.74)	t = -5.89***	d = 0.81
Socioemotional support	-0.24 (1.03)	0.00 (0.81)	t = -1.86	d = 0.23
*Neurocognition*, *M (SD)*				
Global cognition	47.52 (6.89)	50.50 (5.64)	t = -3.46***	d = 0.47
Verbal fluency	49.14 (8.46)	50.62 (6.73)	t = -1.42	d = 0.19
Executive functioning	47.02 (9.05)	51.32 (8.53)	t = -3.58***	d = 0.49
Processing speed	49.06 (8.49)	52.06 (8.28)	t = -2.63**	d = 0.36
Learning	43.47 (8.92)	47.72 (8.76)	t = -3.55***	d = 0.48
Memory	44.28 (9.05)	47.80 (8.43)	t = -2.95**	d = 0.40
Working memory	47.91 (9.14)	49.20 (10.41)	t = -0.98	d = 0.13
Psychomotor speed	49.70 (10.63)	52.71 (10.38)	t = -2.10*	d = 0.29
*Daily functioning*, *mdn [IQR]*				
Functional declines (IADL)	1 [0–3]	0 [0–0]	$\:{\chi\:}^{2}$ = 44.97***	RR = 4.49
Cognitive symptoms (PAOFI)	3 [1–9]	0 [0–1]	$\:{\chi\:}^{2}$ = 103.64***	RR = 5.83

Note. M (SD) = Mean (standard deviation) shown for continuous variables; mdn [IQR] = median [interquartile range] shown for count variables; PWH = people with HIV; PWoH = people without HIV; psych. = psychological; IADL = Instrumental Activities of Daily Living; PAOFI = Patient’s Assessment of Own Functioning Inventory; $\:{\chi\:}^{2}$ = Wald’s chi-square; d = Cohen’s d; RR = incident rate ratio. *p*-values < 0.05 are bolded and denote statistical significance. *p < 0.05, **p < 0.01, ***p < 0.001

### Neurocognition

**Fig. 1:**
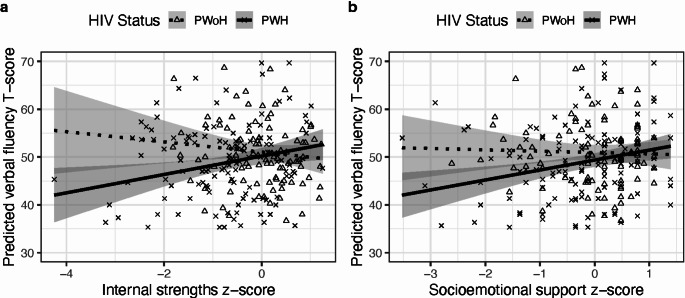
Interaction effects between HIV status and positive psychological factors: (**a**) internal strengths and (**b**) socioemotional support on verbal fluency (higher = better). Higher positive psychological factors were related to better verbal fluency in people with HIV (PWH; solid line), but not in people without HIV (PWoH; dashed line). Shaded region represents 95% confidence intervals

PWH exhibited lower global cognition, executive functioning, processing speed, learning, memory, and psychomotor speed T-scores than PWoH (all *p-values* < 0.05; Table 2). There were significant HIV X PPF interactions, indicating that the relationship between verbal fluency and PPFs differed by HIV status, independent of depressive symptoms (Table 3). Simple main effects analyses showed that PWH with higher internal strengths had higher verbal fluency (B = 2.08, *p* = 0.02), contrary to PWoH (B = -1.21, *p* = 0.22), see Fig. 1a. Similarly, PWH with higher socioemotional support had higher verbal fluency T-scores (B = 2.07, *p* < 0.01), whereas PWoH did not (B = -0.26, *p* = 0.77), see Fig. 1b. All other interactions predicting domain-specific cognition were non-significant (all *p*-values > 0.06).

**Table 3:** HIV status moderates the relationship between positive psychological factors and verbal fluency

*N* = 219			95% CI for B		
Predictor	B	β	Lower	Upper	*p*
Intercept	40.22	0	31.45	48.99	< 0.01***
PWH (ref. PWoH)	-0.84	-0.05	-3.11	1.42	0.46
Internal strengths z-score	-1.07	-0.14	-3.19	1.06	0.32
HIV status X internal strengths	2.98	0.33	0.48	5.48	0.02*
Log CES-D total sum	8.96	0.19	1.49	16.42	0.02*
*Overall model fit: F(4*, *214) = 2.96*, *p* = 0.02, *adjusted R*^*2*^ *= 0.03*					
Intercept	42.73	0	35.05	50.41	< 0.01***
PWH (ref. PWoH)	-1.57	-0.10	-3.70	0.56	0.15
Socioemotional support z-score	-0.28	-0.03	-2.18	1.61	0.77
HIV status X socioemotional support	2.37	0.24	0.06	4.68	0.04*
Log CES-D total sum	6.80	0.15	0.30	13.30	0.04*
*Overall model fit: F(4*, *214) = 3.39*, *p* < 0.01, *adjusted R*^*2*^ *= 0.04*					

Note. PWH = people with HIV, PWoH = people without HIV, CES-D = Center for Epidemiological Studies Depression Scale, B = unstandardized estimate, *β* = standardized estimate, CI = confidence interval. *p*-values < 0.05 are bolded and denote statistical significance. *p < 0.05, ***p < 0.001

Removing non-significant interaction effects from multivariable models (retaining HIV status, PPFs, and depressive symptoms), internal strengths were positively associated with global cognition (B = 1.17, *p* = 0.03), learning (B = 1.64, *p* = 0.03), and memory (B = 1.59, *p* = 0.03). Socioemotional support was positively associated with global cognition (B = 1.15, *p* = 0.01), processing speed (B = 1.50, *p* = 0.02), and psychomotor speed (B = 1.83, *p* = 0.02). In these multivariable models, having HIV remained significantly associated (all *p-values* < 0.03) with worse global cognition, executive functioning, learning, memory, and processing speed (in model with socioemotional support only). In contrast, depressive symptoms were not a significant covariate in any multivariable model (all *p-values* > 0.32). No other main effects of PPFs were found on neurocognition (all *p-values* > 0.15).

### Daily Functioning

**Fig. 2:**
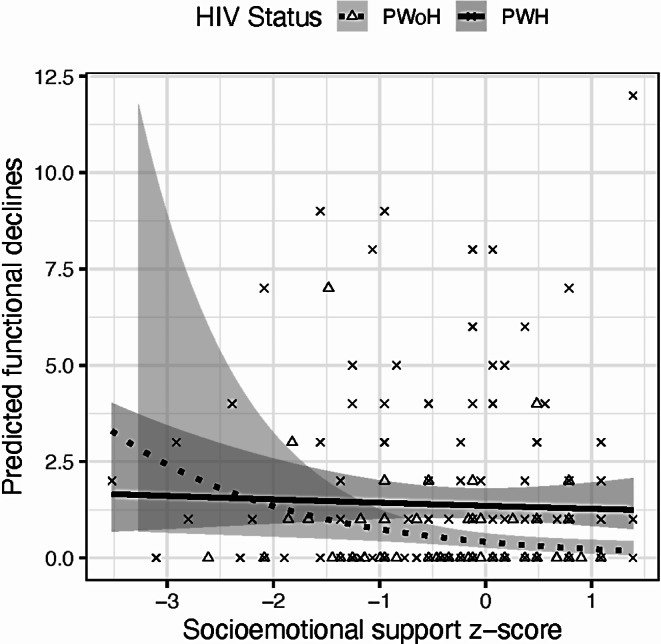
Interaction effects between HIV status and socioemotional support on self-reported functional declines (higher = worse). Higher socioemotional support was related to fewer functional declines in people without HIV (PWoH; dashed line), but not in people with HIV (PWH; solid line). Shaded region represents 95% confidence intervals

PWH reported higher functional declines (1 vs. 0, $\:{\chi\:}^{2}$ = 44.97) and cognitive symptoms (3 vs. 0, $\:{\chi\:}^{2}$ = 103.64) than PWoH (*p-value*s < 0.05; Table 2). Overall, 31.5% of participants attributed functional declines to primarily cognitive vs. physical problems. PWH endorsed this cognitive attribution significantly higher than PWoH (47.4% v.12.4%, $\:{\chi\:}^{2}$ = 32.16, *p* < 0.01). Only higher education was associated with fewer functional declines (rate ratio [RR] = 0.91, *p* < 0.01) below the *p* < 0.15 cut-off; thus, it was the only demographic covariate entered into multivariable regressions. There was a significant HIV X socioemotional support interaction on functional declines (RR = 1.68, *p* = 0.04; Table 4) such that the relationship between functional declines and socioemotional support differed by HIV status, independent of depressive symptoms (RR = 7.36, *p* < 0.01) and education (RR = 0.93, *p* = 0.08). Simple main effects analyses showed that PWoH with higher socioemotional support had fewer functional declines (RR = 0.57, *p* = 0.01), but PWH did not (RR = 0.98, *p* = 0.90), see Fig. 2. No other significant interactions were found on daily functioning (all *p-values* > 0.87).

**Table 4:** HIV status moderates the relationship between socioemotional support and functional declines

*N* = 212			95% CI for RR		
Predictor	B	RR	Lower	Upper	*p*
Intercept	-2.26	0.10	0.02	0.58	0.01*
PWH (ref. PWoH)	1.19	3.29	1.98	5.49	< 0.01***
Socioemotional support z-score	-0.57	0.56	0.36	0.88	0.01*
HIV status X socioemotional support	0.52	1.68	1.01	2.78	0.04*
Log CES-D total sum	2.00	7.36	2.38	22.79	< 0.01***
Education (years)	-0.07	0.93	0.86	1.01	0.08
*Overall model fit: Likelihood ratio, *$\:{\chi\:}^{2}$*(5) = 70.68*, *p* < 0.01					

Note. PWH = people with HIV, PWoH = people without HIV, CES-D = Center for Epidemiological Studies Depression Scale, B = unstandardized estimate, *β* = standardized estimate, CI = confidence interval. *p*-values < 0.05 are bolded and denote statistical significance. *p < 0.05, ***p < 0.001

Removing non-significant interaction effects from multivariable models (retaining HIV status, PPFs, depressive symptoms, and education [in functional decline models only]), higher internal strengths were associated with fewer functional declines (RR = 0.58, *p* < 0.01) independent of having HIV (RR = 2.42, *p* < 0.01), depressive symptoms (RR = 1.62, *p* = 0.48), and education (RR = 0.96, *p* = 0.26). Similarly, higher internal strengths were associated with fewer cognitive symptoms (RR = 0.67, *p* < 0.01) independent of having HIV (RR = 3.13, *p* < 0.01) and depressive symptoms (RR = 9.51, *p* < 0.01). Socioemotional support was not related to cognitive symptoms (RR = 0.96, *p* = 0.69) after accounting for HIV status (RR = 3.97, *p* < 0.01) and depressive symptoms (RR = 25.48, *p* < 0.01).

## Discussion

The current study found that internal strengths and socioemotional support are unique PPFs that have differential relationships with cognition and daily functioning based on HIV status. As hypothesized, PWH had stronger positive associations between PPFs and verbal fluency, independent of depressive symptoms, compared to PWoH. Irrespective of HIV status and depressive symptoms, higher socioemotional support and internal strengths were associated with better global cognition, but PPFs differed in their associated cognitive domains. Higher socioemotional support was related to better processing and psychomotor speed, whereas higher internal strengths were related to better learning and memory. Though having HIV was associated with poorer objective neurocognitive performance in global cognition, executive functioning, learning, memory, and processing speed, depressive symptoms were not.

Contrary to our hypothesis, we did not find PWH to have stronger negative associations between PPFs and daily functioning impairments compared to PWoH. In fact, PWoH with higher socioemotional support had fewer functional declines, but PWH did not. Irrespective of HIV status and depressive symptoms, higher internal strengths were associated with fewer self-reported functional declines and cognitive symptoms. Internal strengths were a better predictor of functional declines than depressive symptoms; however, depressive symptoms were a strong predictor of cognitive symptoms. The finding that depression may bias self-reported cognitive symptoms has been found previously [60, 61]. Having HIV was associated with both greater functional declines and cognitive symptoms.

There are multiple findings in the literature that may explain the positive relationships between PPFs and verbal fluency in PWH, and between PPFs and global cognition in the overall sample. The broaden-and-build theory [62] posits that positive emotions illicit an adaptive “broadened” mindset, increasing behaviors that “build” a wide range of positive psychological resources (e.g., social support) [63]. Accordingly, PWH with greater PPFs may have greater opportunities to engage in lifestyle factors that benefit cognition. Greater lifestyle factors (e.g., physical exercise, social activity) have shown to be incrementally related to decreased rates of HIV-associated NCI [64]. Moreover, PPFs that constitute internal strengths map closely onto eudaimonic factors (e.g., purpose and meaning in life), which are characterized by personal growth, values, and ethics [65]. Multiple meta-analyses and systematic reviews of studies [66–69] conducted among large cohorts of older PWoH (≥ 50 years old) have linked greater purpose and meaning in life with better verbal fluency, episodic memory, and decreased risk of incident dementia by about 20%.

Verbal fluency, specifically, has been associated with social support among PWH. In a recent study, lower verbal fluency was related to worse social support network characteristics, including fewer individuals available for social support related to HIV (e.g., ART adherence) and fewer positive interactions [70]. Thus, impairments in verbal fluency may inhibit cognitive skills required for social support. An optimal level of cognitive functioning, more broadly, may be necessary to recruit and access social support contacts, which can lead to decreased stress and perceived illness-related disability [71, 72]. As such, interventions that bolster PPFs may encourage one to maximize resources that promote better cognition.

There is substantial evidence that increased social support is related to improved HIV-related health outcomes (e.g., increased ART adherence, greater engagement in HIV care) [35–37, 73–75]. The finding that PWH did not have lower daily functioning impairments with greater socioemotional support may be explained by qualitative differences in social support. Definitions of social support in the HIV literature vary widely [76], can be formal (e.g., health care providers) or informal (e.g., friends, family, community), and differ by type (e.g., emotional, informational, instrumental). Furthermore, the current study was limited in that the socioemotional support measures focused on frequency of social engagement and perceived emotional support from others, and not on instrumental support (e.g., others assisting with daily tasks). Though PWH and PWoH did not significantly differ in levels of socioemotional support in the current study, PWH may need greater support from others to accommodate a disproportionately higher degree of functional impairment and higher burden of psychiatric and medical comorbidities.

Social support related to HIV or HIV-specific care (e.g., awareness of HIV status, ART adherence, engagement in healthcare) may be critical for PWH, particularly in the context of HIV-related stigma. For example, PWH are more likely to disclose their HIV status to and perceive greater support from friends than family members [77]. Moreover, PWH with higher social support may have more members in their network who provide HIV-specific support (i.e., aware of HIV status and taking ART) [70]. Types of social support can also depend on the intersection of other sociocultural factors that may be associated with discrimination such as race/ethnicity, sexual minority status, and housing status. For example, Black and Latino PWH are more dependent on formal support for HIV-specific care versus informal networks for more general care (e.g., emotional, household) [78]. In contrast, higher internal strengths were related to improved daily functioning outcomes in the overall sample, which is in line with prior findings linking internal PPFs (e.g., self-efficacy, optimism) to improved HIV-related outcomes and greater engagement in HIV care [34, 35, 79, 80]. These outcomes may also reflect greater access to healthcare (e.g., reliable transportation, insurance) [81] and maintenance of overall health. These findings suggest that increasing internal strengths as opposed to socioemotional support may be more promising targets for improving daily functioning.

PPFs also demonstrated unique relationships with cognition and daily functioning for the total sample. Our findings add to the accumulating body of literature linking socioemotional support and internal strengths to better cognition. In our sample, higher socioemotional support was related to better processing speed and psychomotor speed. Though some studies have found a positive relationship between social support and processing speed, the benefits of various forms of social support on domain-specific cognition remain unclear [72, 82]. Higher internal strengths were related to better learning and memory, which is also consistent with prior work demonstrating relationships between these domains and grit [27] and perceived control [25]. Grit and perceived control may overlap with memory self-efficacy (MSE). MSE has been described as the confidence in one’s perceived memory capabilities, which tends to decline with age [83]. Higher MSE leads to greater task persistence, which increases the likelihood of better episodic memory ability [84]. As many daily tasks involve memory, greater perceived competency may engender persistence that promotes independent functioning in older age.

### Limitations

The current study has limitations. Due to the cross-sectional nature of the analyses, we were not able to determine if PPFs buffered the effect of subsequent NCI and/or functional impairment. PWoH were generally healthy and had few self-reported daily functioning impairments, which could have influenced the findings. Lastly, we did not emphasize recruitment of geographically, ethnically, and demographically diverse PWH that reflect the diversity of PWH. Participants were mostly male, White, relatively well-controlled in their HIV disease, and are healthy and functional enough to participate in research; thus, our sample is not representative of all PWH. Future studies should focus on people from racially/ethnically minoritized populations.

In summary, the current study contributes to an important gap in the literature by examining unique groups of PPFs and their relationships to cognition and daily functioning. Among PWH, internal strengths were more strongly related to better cognition (verbal fluency) than PWoH. On the other hand, greater socioemotional support was related to less functional decline in PWoH, but not in PWH. If confirmed, the results suggest that interventions that strengthen grit, optimism, life satisfaction, positive attitudes toward aging, or personal mastery may protect against cognitive decline in PWH. Interventions focused on strengthening socioemotional support among PWH may need further investigation.

## Electronic Supplementary Material

Below is the link to the electronic supplementary material.

Supplementary Material 1

## Data Availability

Contact corresponding author.
