# Supplementary material for: Positive Psychological Factors Relate to Domain-Specific Cognition and Daily Functioning in Middle-Aged and Older Adults with HIV
Source: AIDS Behav. 2025 Mar 3;29(5):1669–80. doi: 10.1007/s10461-025-04636-8 (PMC12031858; doi:10.1007/s10461-025-04636-8)
Supplement: Supplementary file 1 — Supplementary Material 1 [file 10461_2025_4636_MOESM1_ESM.docx]

| **Supplementary Table 1** Positive psychological factor measures | | |
| --- | --- | --- |
| **Measure**  **(number of items)** | **Construct, *example item***  **(response format)** | **Directionality**  **(score: range)** |
| *Internal strengths factor measures:* | | |
| Grit Scale [44]  (12-item short-form) | Grit (perseverance of long-term goals despite setbacks)  e.g., *“Setbacks don’t discourage me”*  (1-not like me at all to 5-very much like me) | Higher score = grittier  (overall average: 1-5) |
| Philadelphia Geriatric Center Morale Scale [45] (5-item attitude toward own aging subscale) | Attitude toward aging (attitude toward and evaluation of the aging process one experiences)  e.g., *“I am as happy as when I was younger”*  (0-disagree and 1-agree) | Higher score = more positive attitude toward aging  (sum: 0-5) |
| Life Orientation Test-Revised [46] (6-item overall resilience) | Optimism (individual differences in generalized optimism vs. pessimism)  e.g., *“I’m always hopeful about my future”*  (1-strongly agree to 5-strongly agree) | Higher score = more optimism  (sum: 6-30) |
| Satisfaction with Life Scale [47] (5-item) | Life satisfaction (global cognitive judgments of satisfaction with one’s life and participant well-being)  e.g., *“I am satisfied with my life”*  (1-not at all to 4-strongly agree) | Higher score = more satisfaction  (sum: 5-35) |
| Pearlin Personal Mastery Scale [48] (7-item) | Personal mastery (self-concept and the extent to which one perceives themself in control of forces that significantly impact their life)  e.g., *“What happens to me in the future mostly depends on me”*  (1-strongly disagree to 4-strongly agree) | Higher score = more personal mastery  (sum: 7-28) |
| *Socioemotional support factor measures:* | | |
| Emotional Support Scale [26] (2-item emotional support subscale) | Emotional support (frequency and availability of emotional support)  e.g., *“How often are your spouse, children, close friends, and/or relatives willing to listen when you need to talk about your worries or problems?”*  (1-never to 4-frequently) | Higher score = more emotional support  (average: 0-3)^a^ |
| Duke Social Support Index [49] (4-item social interaction subscale) | Social support (number of close relationships and frequency of socialization)  e.g., *“About how often did you go to meetings or clubs, religious meetings, or other groups that you belong to in the past week?”*  (1-none to 3-seven or more times) | Higher score = more social support  (sum: 4-12) |
| *Note.* Table adapted from Ham et al., 2023 [43]. ^a^Emotional Support Scale was administered with a response scale from 1 to 4, but the average score was calculated using a range from 0 to 3. | | |

| Supplementary Table 2 Neuropsychological battery | |
| --- | --- |
| **Cognitive domain** | **Test** |
| Verbal fluency | Controlled Oral Word Association Test [50]: Category Fluency (Animals)  Controlled Oral Word Association Test [50]: Letter Fluency (FAS) |
| Executive functioning | Wisconsin Card Sorting Test-64 (perseverative responses) [50, 51] / Stroop Color and Word Test (interference score) [51]  Trail Making Test: Part B [50] |
| Processing speed | Wechsler Adult Intelligence Scale-III (WAIS-III) [52]: Digit Symbol Coding  WAIS-III: Symbol Search [52]  Trail Making Test: Part A [50]  Stroop Color and Word Test (color score) [51] |
| Learning | Hopkins Verbal Learning Test-Revised [51, 53] (trials 1-3)  Brief Visuospatial Memory Test-Revised [51, 54] (trials 1-3) |
| Memory | Hopkins Verbal Learning Test-Revised [51, 53] (delayed recall)  Brief Visuospatial Memory Test-Revised [51, 54] (delayed recall) |
| Attention/Working memory | WAIS-III Letter-Number Sequencing [52],  Paced Auditory Serial Addition Task [55] (first channel only) |
| Psychomotor speed | Grooved Pegboard Test [50] (dominant and non-dominant hands) |
| *Note.* Tests used to derive demographically adjusted (age, gender, education, race) global and domain-specific T-scores. | |
